# Glutamine and norepinephrine in follicular fluid synergistically enhance the antioxidant capacity of human granulosa cells and the outcome of IVF-ET

**DOI:** 10.1038/s41598-022-14201-1

**Published:** 2022-06-15

**Authors:** Lulu Wang, Chengliang Zhou, Junyan Sun, Qiuwan Zhang, Dongmei Lai

**Affiliations:** 1 The International Peace Maternity and Child Health Hospital, School of Medicine, Shanghai Jiao Tong University, 145, Guang-Yuan Road, Shanghai, 200030 China; 2 Shanghai Key Laboratory of Embryo Original Diseases, Shanghai, 200030 China

**Keywords:** Genetics, Medical research

## Abstract

An increasing number of studies demonstrate that changes in neurotransmitters metabolic levels in follicular fluid are directly related to oocyte maturation, fertilization, the quality of embryo and pregnancy rates. However, the relationship between the intra-follicular neurotransmitters and the function of granulosa cells (GCs), and the outcome of in vitro fertilization-embryo transfer (IVF-ET) is not clear. Human follicular fluid and cumulus GCs were harvested from large follicles obtained from patients undergoing IVF. Neurotransmitters and steroid hormones in follicular fluid were measured through liquid chromatography-tandem mass spectrometry (LC–MS/MS) and high-performance liquid chromatography-mass spectrometry (HPLC–MS/MS). Based on the content of glutamine (Gln) in follicular fluid, the samples were divided into two groups: high Gln level group and low Gln level group. The expression of proliferation-, steroidogenesis- and antioxidant-related genes in GCs was detected by qRT-PCR. In vitro, KGN cells were used to further verify the effects of Gln and NE on GCs function. Primary and secondary outcomes were the number of mature and retrieved oocytes, and the ratio of high-quality embryos, respectively. Gln (46.75 ± 7.74 μg/mL) and norepinephrine (NE, 0.20 ± 0.07 μg/mL) were abundant neurotransmitters in follicular fluid, and exhibited a significantly positive correlation (R = 0.5869, P < 0.005). In high Gln level group, the expression of proliferation, steroidogenesis and antioxidant-related genes in GCs were higher than those in low Gln level group, and the contents of estriol and E2 in follicular fluid were more abundant. Moreover, the concentrations of Gln and NE in follicular fluid showed significantly positive correlation with *IDH1* expression in GCs (R = 0.3822, R = 0.4009, P < 0.05). Importantly, a significantly positive correlation was observed between *IDH1* expression in GCs and the ratio of higher-quality/cleaved embryos (R = 0.4480, P < 0.05). In vitro studies further demonstrated that Gln and NE played synergistically function in improving GCs proliferation and E2 production by upregulating IDH1 expression. These data demonstrate that Gln and NE in follicular fluid might play significant positive roles in GCs function, and may be potential predictors for selecting optimal quality oocytes and evaluating the quality of embryonic development.

## Introduction

Infertility is a reproductive system disease characterized by failure to establish a clinical pregnancy after normal unprotected sexual intercourse for 12 months or more, or impairment of the reproductive capacity of an individual or with his or her partner^1,2^. Infertility has been recognized as a worldwide public health problem by the World Health Organization (WHO), and approximately half of these cases lack an definitive explanation for pregnancy failure^3^. It is estimated that in western countries, one in six couples has infertility, which is recognized as the cause of psychological distress in both men and women^4^. In vitro fertilization (IVF) offers a possibility for achieving pregnancy, but the success rate is still suboptimal^5,6^.

As an important biofluid, follicular fluid provides a special microenvironment for follicular development and impacts oocyte quality, implantation and early embryo development. Follicular fluid is rich in proteins, including sex hormones, nutrient substances, cytokines, growth factors and neurotransmitters, which is critical for oocyte development^7^. Cumulus GCs represent the predominant somatic cell type of follicle development and are involved in steroidogenesis and folliculogenesis. Furthermore, they connect with oocytes via direct gap junctions to support the developing oocyte^8–10^. The process of follicular development involves local biochemical exchanges and substantial modifications in cellular metabolism.

Amino acids serve as substrates for the synthesis of proteins, not only enhance embryonic development but also affect blastocyst formation in mice. A study has demonstrated that Glycine (Gly), Glutamine (Gln), alanine (Ala), Glutamate (Glu) and Proline (Pro) are the most abundant amino acids in follicular fluid^11^. The disturbance of follicular amino acid metabolism is related to women with polycystic ovary syndrome (PCOS), which may be the reason for the poor pregnancy outcome in obese patients and increased risk of abortion in PCOS patients^12^. In addition, they are not only components of proteins, but also regulate neurotransmitter homeostasis, such as a metabolite shuttle known as the glutamate/GABA-glutamine cycle describes the release of neurotransmitter glutamate or GABA from neurons and subsequent uptake into astrocytes, play import roles in nervous system^13,14^. However, whether the neurotransmitters metabolic levels provided by follicular fluid influences the function and hormone synthesis of cumulus GCs need to be further investigated.

Oxidative stress plays an important role in reproductive processes, including follicular development, oocyte maturation, ovulation and fertilization, as well as embryo implantation and embryo development^15^. Excessive generation of ROS, if not efficiently counterbalanced by antioxidative enzymes, may cause damage to oocytes and GCs, leading to poor oocyte quality^9^. In our previous study, psychological stress-induced oxidative stress decreased the expression of the antioxidant gene IDH1 to disturb and compromise the function of GCs, accelerating ovarian senescence^16^. Antioxidant supplementation in IVF culture medium could increase fertilization rates and subsequent healthy embryo development^17^. Previous studies reported that NE and its metabolites could induce the generation of reactive oxygen species (ROS) in human GCs^18^. Once the level of NE concentration exceeds the physiological concentration, it may lead to ROS-related pathophysiology events including oxidative stress and cell death^19^. However, little is known about the impact of neurotransmitters in follicular fluid on oxidative capacity of human GCs.

During the process of follicle growth to ovulation, which is mediated by the preovulation production of gonadotropins, metabolic profiles change dynamically in follicular fluid. Thus, a better understanding of the neurotransmitters metabolic levels in follicular fluid is important for exploring the metabolic cooperativity between oocytes and cumulus GCs.

In this study, we quantified the neurotransmitters metabolites and steroid hormones in follicular fluid from IVF patients by LC–MS/MS and HPLC–MS/MS technology, and evaluated the impact of Gln and NE in follicular fluid on the function and antioxidative capacity of cumulus GCs, which could be related with the clinical outcome of IVF patients. Additional in vitro cell experiments were performed to further explore the effects of Gln and NE on the proliferation and steroidogenesis of GCs.

## Methods

### Study design

This is an observational study of infertile women before and during IVF treatment at the International Peace Maternity and Child Health Hospital (IPMCH), Shanghai, China. Follicular fluid and cumulus GCs on the day of oocyte retrieval were obtained from 27 patients undergoing IVF at the reproductive center. The subjects were recruited with male factor infertility or tubal factor infertility. Subjects with endometriosis and PCOS were excluded. The age of the patients ranged from 25 to 40 years, and the size of the follicles ranged from 19 to 24 mm.

### Collection of GCs and follicular fluid

The size of the follicle was estimated by ultrasound at the time of oocyte retrieval. Follicular aspirates contain oocytes surrounded by cumulus GCs. After the oocytes were removed by the embryologist, the remaining material was centrifuged at 3000 g for 15 min to isolate the GCs. The fluid from the first aspirated follicle was used to measure hormone and neurotransmitter metabolic levels.

### Embryo quality assessment

Embryos were assessed at Day 3 based on the Istanbul consensus on embryo assessment^20^, and higher quality embryos were defined as Day 3 embryos that had even cleavage, 7–9 blastomeres and < 10% fragments.

### Quantification of neurotransmitters metabolites in follicular fluid by liquid chromatography–tandem mass spectrometry (LC–MS/MS)

Human follicular fluid was prepared for neurotransmitters metabolomics detection. Twenty-three metabolites (Ach: Acetylcholine chloride; Gln: Glutamine; Glu: Glutamate; His: L-Histidine; NE: norepinephrine; Tyr: Tyrosine; Trp: Tryptophan; Kyn: Kynurenine; GABA: 4-Aminobutyric acid; HisA: Histamine; PA: Picolinic acid; TyrA: Tyramine; DA: Hydroxytyramine hydrochloride; TrpA: Tryptamine; 5-HT: Serotonin hydrochloride; E: Adrenaline hydrochloride; KynA: Kynurenic acid; 5-HIAA: 5-Hydroxyindole-3-acetic acid; DOPA: Levodopa; XA: Xanthurenic acid; VWA: Vanillylmandelic Acid; 5-HTTP: 5-Hydroxytryptophan; MT: Melatonin) and the internal standards (Tryptophan-D3) were analyzed using LC–MS/MS as described previously^21,22^ by BioNovo Gene Co., Ltd (Suzhou, China).

In brief, LC–MS/MS was conducted by A Waters ACQUITY UPLC coupled with an AB 4000 triple quadrupole mass spectrometer (AB SCIEX, USA) using a Waters ACQUITY UPLC BEH C18 column (2.1 mm × 100 mm, 1.7 μm, Waters, USA). The Mobile phase A consisted of methanol and water with 0.1% formic acid (1:9, v/v), whereas mobile phase B consisted of methanol and water with 0.1% formic acid (1:1, v/v). The LC gradient elution, was at a flow rate of 0.3 mL/min at 0–8.0 min and 0.4 ml/min at 8.0–17.5 min, was 10%-30% B at 0–6.5 min; 30%∼100% B at 6.5–7 min; 100% B at 7–14 min; 100%-10% B at 14–17.5 min. The injection volume was 5 μL and column temperature was set to 40 °C. Twenty-three metabolites were simultaneously reported in LC–MS/MS multiple reaction monitoring (MRM) mode. Data analysis was performed with the Analyst 1.6 software (AB SCIEX, USA).

### RNA extraction and qRT-PCR

GCs were collected in TRIzol reagent (Invitrogen, Carlsbad, CA, USA), and total RNA was extracted according to previously described methods^16^. A total of 1 μg RNA was converted to cDNA with a Takara kit (Applied Biosystems Foster City, CA, USA; Takara, Shiga, Japan). The genes of interest were amplified with a 7900HT fast real-time PCR system (Applied Biosystems) and SYBR Green Real-time PCR Master Mix (Applied Biosystems/Takara). The PCR primers were designed according to the cDNA sequences in the NCBI database. The primer sequences used are shown in Supplementary Table 1. The cycling conditions used for the PCR analysis were as follows: 95 °C for 5 s, 60 °C for 30 s, and 72 °C for 30 s (40 cycles). 18S was used as an internal control. The 2^-ΔΔCt^ method was employed to determine the relative mRNA expression level.

### Hormone metabolic levels in follicular fluid

The concentrations of twenty kinds of hormones in follicular fluid were detected by high-performance liquid chromatography–mass spectrometry (HPLC–MS/MS) as described previously^23^ by BioNovo Gene Co., Ltd. (Suzhou, China). Briefly, an Agilent 1200 series high-performance liquid chromatography (HPLC) instrument (Agilent, USA) was utilized. A PAL autosampler (CTC, Swiss) and a Gemini-NX-C18 column (2.0 mm × 50 mm, 3 μm, Waters, USA) were also used. The ion source was a quadrupole electrostatic field orbit trap high-resolution mass spectrometer API-4000 (Applied Biosystems, USA). The scanning mode was multiple reaction monitoring (MRM). In addition, the concentration of testosterone and cortisol in follicular fluid was detected by the chemiluminescence method^24^.

### Human granulosa tumor cell line (KGN) culture and treatment

The human ovarian granulosa cell line was kindly gifted by Dr. Zuwei Yang (IPMCH, School of Medicine, Shanghai Jiao Tong University, Shanghai, China). KGN cells firstly were cultured in the glutamine deprived medium (Advanced DMEM/F12 media, Gibco, 12634010) supplemented with 10% fetal calf serum and antibiotics (100 IU/mL penicillin, 100 μg/mL streptomycin) in a 5% CO_2_ atmosphere at 37 °C for 24 h in order to consume glutamine stored in cells. And then, glutamine and NE were added into the culture medium of KGN cells for another 48 h. NE (HY-13715, MCE, NJ, USA) was solved in dimethyl sulfoxide (DMSO, Yeasen, shanghai, China) at storage concentrations of 100 mM, and treated cells at a concentration of 10 μM diluted with the advanced DMEM/F12 media. Glutamine (HY-N0390, MCE, NJ, USA) treated cells at a concentration of 2.5 mM or 5 mM diluted with the advanced DMEM/F12 media.

### Enzyme-linked immunosorbent assay (ELISA)

Cell culture supernatant samples were collected and centrifuged for 20 min at 1000×*g* at − 4 °C. The supernatant was stored at − 80 °C for further analysis. The concentrations of 17 β-estradiol and progesterone in cell culture supernatant were measured by ELISA kits (Xinle Biological Technology, Shanghai, China), according to the manufacturer’s instruction.

### Western blotting

Proteins were extracted from cultured cells with RIPA lysis buffer (Yeasen, Shanghai, China) and protein concentration was measured via a BCA kit (Thermo Scientific, Rockford, IL, USA). The protocol for western blotting was reported previously^16^. The following primary antibodies were used: rabbit antibodies against β-tubulin (1:1000, CST, MA, USA), PCNA (1:1000, CST, MA, USA) and IDH1 (1:1000, Abcam, Cambridge, UK). After incubation with primary antibodies at 4 °C overnight, the membranes were incubated with horseradish peroxidase-conjugated secondary antibodies (1:2500, CST, MA, USA) for 1 h at room temperature. The bands were detected by electrochemiluminescence (ECL, Millipore, Billerica, MA, USA). The relative intensity of the target proteins was normalized to β-tubulin using ImageJ software (NIH, Bethesda, MD, USA).

### EdU staining

The proliferation of KGN cell was evaluated by EdU staining (RiboBio, Guangdong, China). Briefly, EdU was added to the cell culture medium at a concentration of 10 µM. Cells were cultured for 24 h and stained with Alexa 594 and DAPI. Images were captured under a microscope (Leica, WetzlaR, Germany) and positive fluorescence signals were counted. The number of EdU-positive cells per 100 cells was evaluated.

### Statistical analysis

All statistical analyses were performed using Prism software, and a *p* value of < 0.05 was considered statistically significant. Means and standard error were used as descriptive statistics. T-tests were used to compare different variables between the low-level and high-level glutamine groups. Pearson's correlation coefficients and corresponding 95% confidence intervals (CIs) were used to assess the correlation between neurotransmitter metabolites in follicular fluid, and the correlation between IDH1 expression in GC and Gln/NE in follicular fluid. Multiple linear regressions were used to test the correlation between Gln/NE in follicular fluid and the ratio of high-quality embryos, as well as IDH1 expression in GC and the ratio of high-quality embryos, adjusting for spouse age, sperm density, and sperm motility. In the current study, when a power analysis was performed with 80% power and an α value of 0.05, the number of samples in each group needed to be 13 to confirm statistical significance.

### Ethics approval and informed consent

Informed consents for participating in this study were obtained from all the patients and this study was approved by the Institutional Ethics Committee of the International Peace Maternity and Child Health Hospital (Ethical statement number: (GKLW) 2018-43). All experiments were performed in accordance with the relevant guidelines and regulations.

### Consent for pubication

All authors have agreed to publish this article.

## Results

### Glutamine is positively correlated with norepinephrine content in follicular fluid of IVF patients

**Figure 1:**
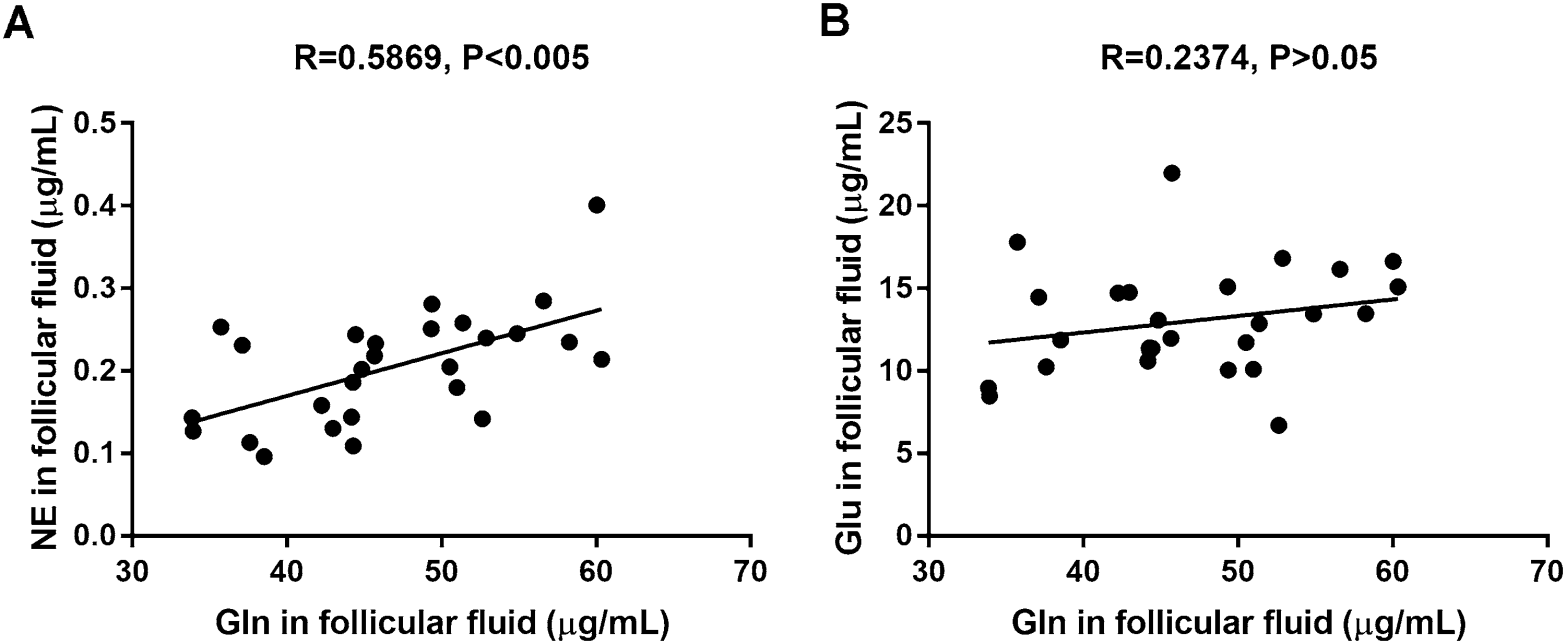
Correlation analysis of neurotransmitter concentrations in human follicular fluid on the day of oocyte retrieval. (**A**) A positive correlation between the concentration of glutamine (Gln) and norepinephrine (NE) in follicular fluid (n = 27, R = 0.5869, P < 0.005). (**B**) There was no correlation between the Gln and Glutamate (Glu) concentrations in the follicular fluid (n = 27, R = 0.2374, P > 0.05).

Dynamic neurotransmitters metabolic changes within follicular fluid are important for cumulus expansion, which represents the consequence of oocyte cumulus complex (OCC) metabolic cooperation^25,26^. In this study, we first measured the content of neurotransmitters metabolites in follicular fluid by LC–MS/MS. The results regarding the concentrations of Ach (0.02 ± 0.01 μg/mL), Gln (46.75 ± 7.74 μg/mL), Glu (13.00 ± 3.27 μg/mL), His (1.30 ± 0.49 μg/mL), NE (0.20 ± 0.07 μg/mL), Tyr (6.54 ± 2.91 μg/mL), Trp (6.54 ± 1.33 μg/mL), Kyn (0.31 ± 0.12 μg/mL) in human follicular fluid are shown in Table 1. The other fifteen neurotransmitters were not detected in most of the follicular fluid samples. Pearson’s correlation analysis showed that Gln was positively correlated with the NE content in follicular fluid (R = 0.5869, P < 0.005, Fig. 1A). However, there was no relationship between the contents of Gln and Glu in follicular fluid (R = 0.2374, P > 0.05, Fig. 1B).

**Table 1:** Concentration of multiple neurotransmitters in human follicular fluid.

Sample ID	Ach	Gln	Glu	His	NE	Tyr	Trp	Kyn
	(ug/ml)	(ug/ml)	(ug/ml)	(ug/ml)	(ug/ml)	(ug/ml)	(ug/ml)	(ug/ml)
01	0.019	45.715	21.989	1.083	0.233	6.431	7.227	0.238
02	0.025	49.37	10.045	1.472	0.281	6.231	7.373	0.548
03	0.080	60.028	16.639	3.620	0.401	19.822	5.658	0.757
04	0.018	49.313	15.104	1.365	0.251	4.751	6.977	0.307
05	0.019	52.869	16.819	1.510	0.240	5.724	7.711	0.350
06	0.016	54.879	13.450	1.273	0.245	6.297	5.830	0.202
07	0.019	35.712	17.795	1.074	0.253	5.903	6.359	0.361
08	0.027	44.439	11.364	1.389	0.244	6.018	4.618	0.307
09	0.018	51.359	12.867	1.329	0.258	6.633	6.925	0.319
10	0.023	60.335	15.089	0.947	0.214	7.698	6.458	0.243
11	0.020	44.826	13.077	1.046	0.202	4.362	5.838	0.284
12	0.026	42.962	14.758	1.339	0.130	5.837	5.290	0.272
13	0.021	58.259	13.474	1.411	0.235	5.413	5.804	0.225
14	0.017	56.594	16.155	1.208	0.285	7.238	10.141	0.241
15	0.02	52.631	6.706	1.455	0.142	10.169	9.157	0.255
16	0.018	44.161	10.595	1.417	0.144	5.729	6.591	0.455
17	0.017	37.091	14.466	1.146	0.231	4.984	7.835	0.346
18	0.021	33.855	8.973	1.014	0.143	4.525	5.623	0.261
19	0.016	50.998	10.091	1.029	0.180	6.335	8.394	0.243
20	0.014	44.261	11.385	1.012	0.186	4.101	6.881	0.284
21	0.018	50.524	11.722	1.249	0.205	6.630	5.096	0.199
22	0.018	38.522	11.873	1.055	0.096	5.849	4.797	0.265
23	0.017	37.578	10.232	1.099	0.113	4.864	5.351	0.248
24	0.018	42.237	14.710	1.106	0.158	6.031	5.536	0.323
25	0.027	44.286	11.252	1.313	0.109	5.851	6.290	0.262
26	0.022	45.651	11.975	1.157	0.218	7.406	7.377	0.336
27	0.026	33.927	8.471	1.103	0.127	5.956	5.352	0.348

*Ach* acetylcholine chloride, *Gln* glutamine, *Glu* glutamate, *His* l-Histidine, *NE* norepinephrine, *Tyr:*tyrosine, *Trp* tryptophan, *Kyn* Kynurenine.

Gln serves as a substrate for the synthesis of proteins in follicular fluid, which directly promotes oocyte nuclear maturation and enhances embryo development^11^. However, the effect of Gln and NE in follicular fluid on the function of cumulus GCs is still unclear. To further investigate the function of Gln and NE in follicular fluid, 27 samples were divided into two groups according to their concentration of Gln, the high Gln level group (> 45 μg/mL) and low Gln level group (< 45 μg/mL).

### Effect of intra-follicular glutamine/norepinephrine on the function of granulosa cells

**Figure 2:**
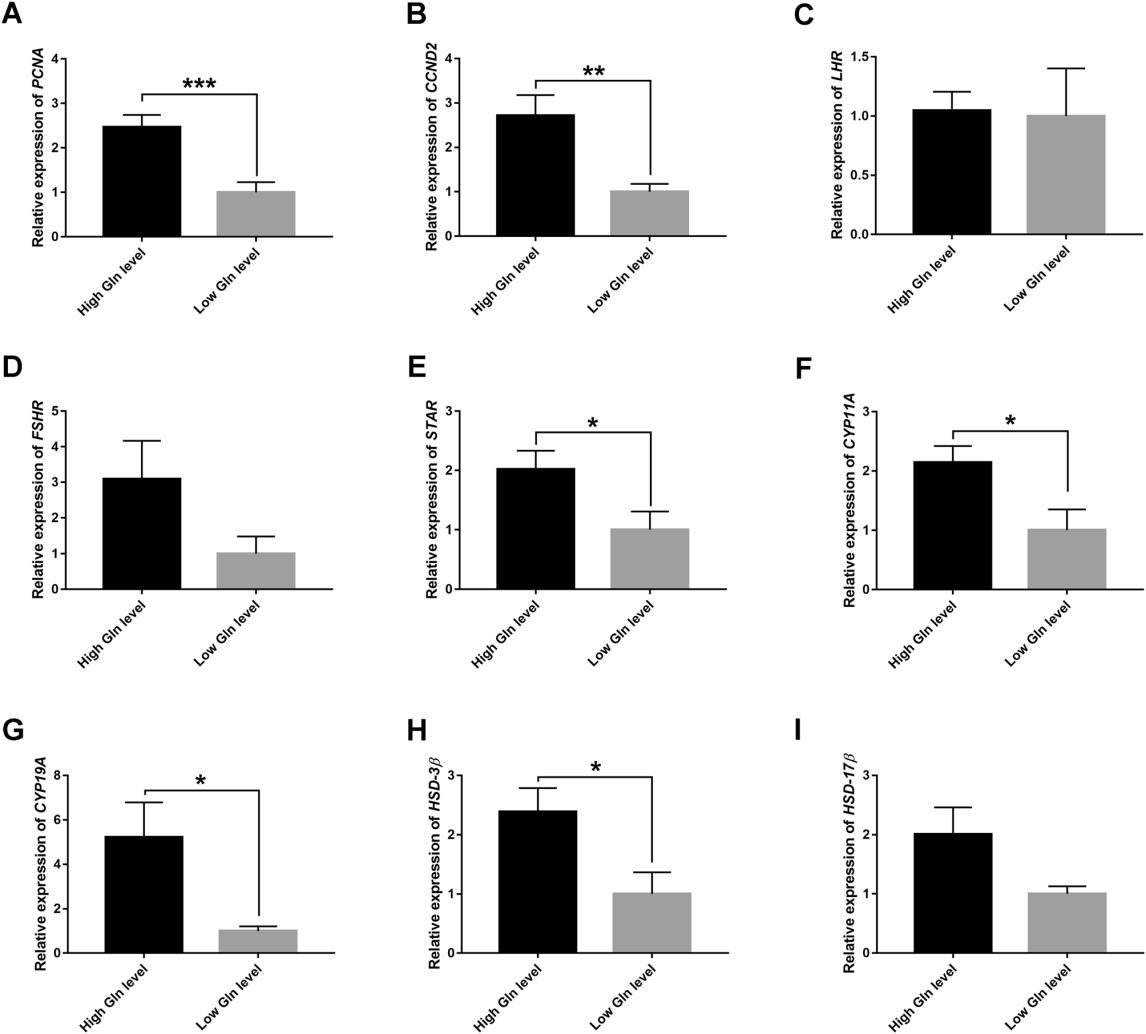
The mRNA expression of proliferation, gonadotropin receptor and sex steroid hormone synthesis-related genes in cumulus granulosa cells between high and low glutamine (Gln) level groups. (**A**,**B**) The column charts show the PCNA and CCND2 mRNA expression in granulosa cells (GCs) in the two groups. (**C**,**D**) The column charts display the LHR and FSHR mRNA expression in the two groups. (**E**–**I**) The mRNA expression of sex steroid hormone synthesis-related genes in the two groups, including STAR, CYP11A, CYP19A, HSD-3β and HSD-17β. Data represent the mean values ± standard error of the mean (mean ± SEM). High Gln level group, n = 14; Low Gln level group, n = 13. All indicated *P* values were determined by Student's t-test. *P value < 0.05; **P value < 0.01; ***P value < 0.001.

The function of cumulus GCs involved in proliferative capacity, receptor expression and steroid hormone synthesis was determined by qRT-PCR. Compared with the low Gln level group, those with high Gln level had a significant twofold increase in *PCNA* and cyclin D2 (*CCND2*) mRNA levels in the GCs (P < 0.001 and P < 0.01, Fig. 2A,B). However, there were no statistically significant differences in luteinizing hormone receptor (*LHR*) or follicle stimulating hormone receptor (*FSHR*) mRNA levels between the two groups (P > 0.05, Fig. 2C,D). Notably, the mRNA levels of the steroid hormone synthesis-related genes, including steroid acute regulatory protein *(STAR),* cytochrome P450 *(CYP11A, CYP19A)* and hydroxysteroid dehydrogenase-3 beta (*HSD-3β*) significantly decreased in the low Gln level group (P < 0.05, Fig. 2E–H). However, there was no significant difference in hydroxysteroid dehydrogenase-17 beta (*HSD-17β* mRNA levels between the two groups (P > 0.05, Fig. 2I). These data demonstrated that the proliferative ability and steroidogenesis of cumulus GCs increased with the high contents of Gln/NE in follicular fluid.

### Glutamine/norepinephrine promote estrogen synthesis in follicular fluid of IVF patients

**Figure 3:**
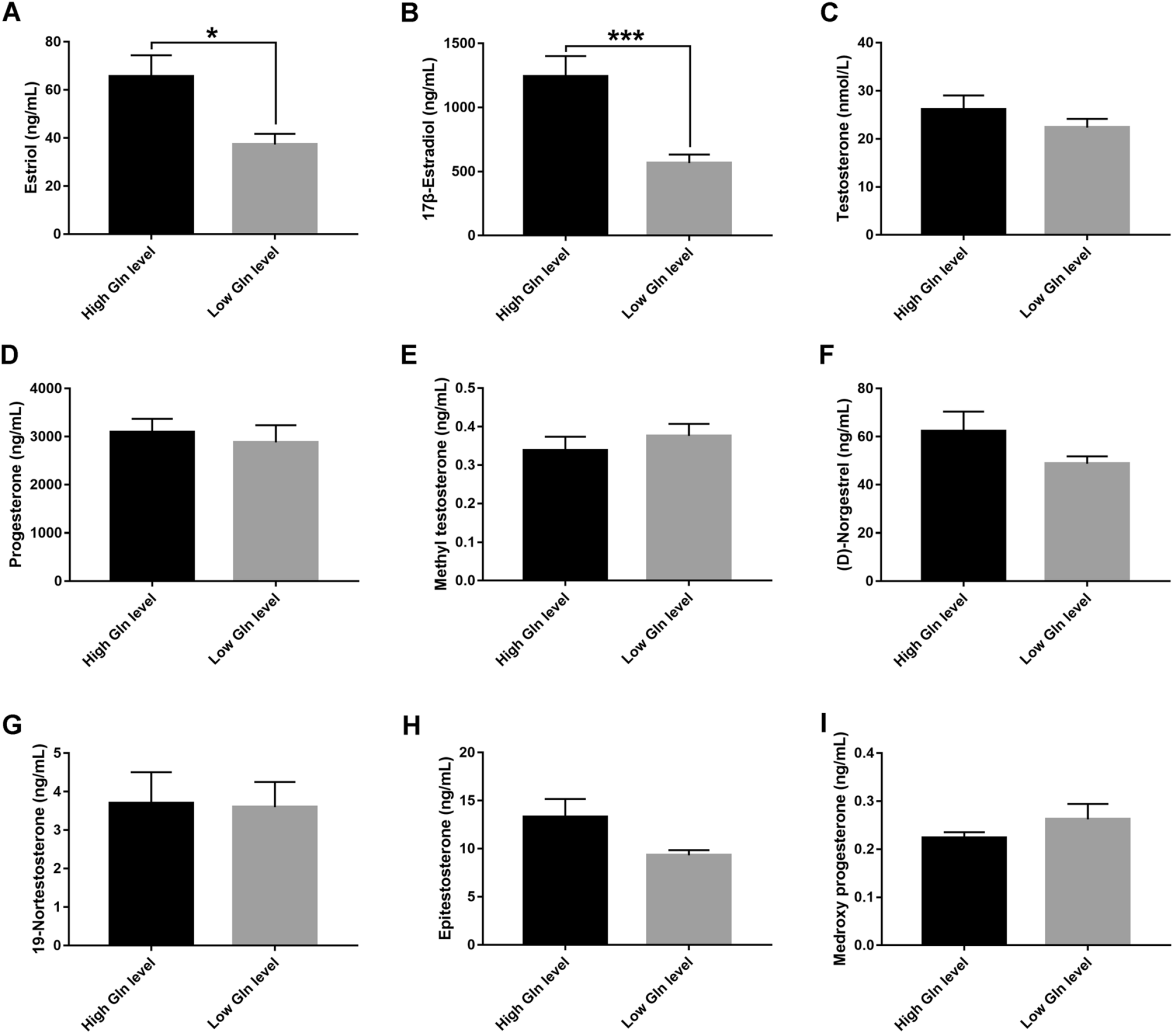
Metabolic levels of steroid hormones measured by HPLC–MS/MS in human follicular fluid. The graphs show kinds of hormone concentrations in human follicular fluid between high and low glutamine (Gln) level groups, including estriol (**A**), 17β-estradiol (**B**), progesterone (**D**), methyl testosterone (**E**), (D)-norgestrel (**F**), 19-nortestosterone (**G**), epitestosterone (**H**) and methoxy progesterone (**I**). (**C**) The column chart shows the concentration of testosterone in the follicular fluid in the two groups, which was measured by a chemiluminescence immunoassay. Data represent the mean values ± standard error of the mean (mean ± SEM). High Gln level group, n = 14; Low Gln level group, n = 13. All indicated *P* values were determined by Student's t-test. *P value < 0.05; ***P value < 0.001.

To obtain an overview of hormone metabolic characteristics in follicular fluid between high and low Gln level groups, hormone profiling was conducted using HPLC–MS/MS. Nine kinds of hormones were detected in the two groups, including estrone, estriol, 17β-estradiol, progesterone, methyl testosterone, (D)-norgestrel, 19-nortestosterone, epitestosterone and methoxy progesterone. In addition, the concentration of testosterone in follicular fluid was measured by a chemiluminescence immunoassay. The results demonstrated that the concentrations of estriol and 17β-Estradiol were significantly higher in the high Gln level group than in the low Gln level group (P < 0.05 and P < 0.001, Fig. 3A,B). There were no statistically significant differences in other hormone contents between the two groups (P > 0.05, Fig. 3C–I). These results revealed that Gln and NE in follicular fluid may play important roles in estrogen synthesis and the production.

### Glutamine/norepinephrine in follicular fluid increase the expression of antioxidant genes in granulosa cells

**Figure 4:**
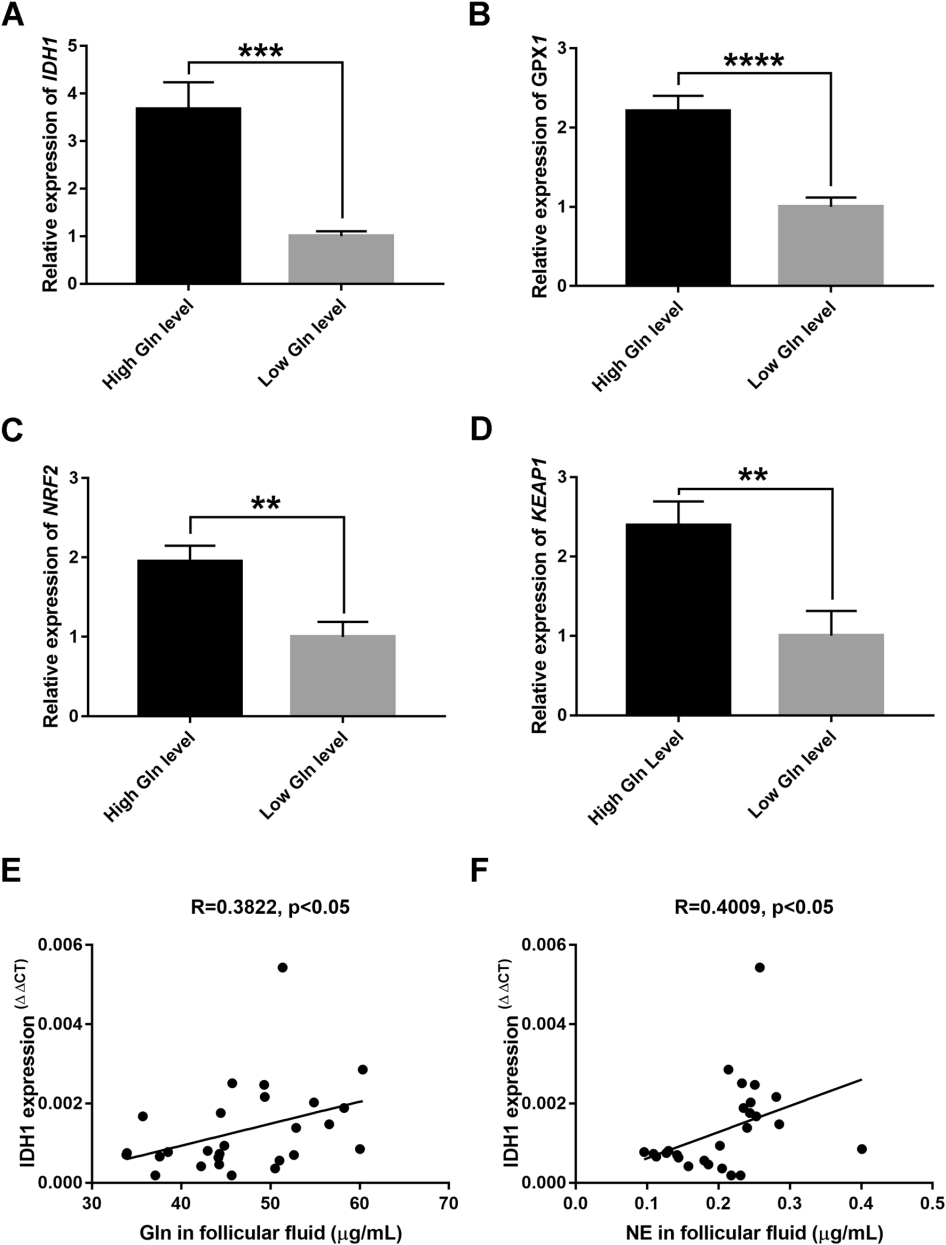
The expression levels of the antioxidative genes in the cumulus granulosa cells between high and low glutamine (Gln) level groups. (**A**–**D**) The column charts demonstrate the expression of antioxidant genes in the two groups, including IDH1, GPX1, NRF2, and KEAP1. (**E**) Correlation analysis of Gln concentration in follicular fluid and IDH1 mRNA expression in cumulus granulosa cells (GCs) (n = 27, R = 0.3822, P < 0.05). (**F**) Correlation analysis of norepinephrine (NE) concentration in follicular fluid and IDH1 mRNA expression in cumulus GCs (n = 27, R = 0.4009, P < 0.05). Data represent the mean values ± standard error of the mean (mean ± SEM). High Gln level, n = 14; Low Gln level, n = 13. All indicated *P* values were determined by Student's t test. **P value < 0.01; ***P value < 0.001; ****P value < 0.0001.

Oxidative metabolism is the major oxygen consumer in the follicle, which could be affected by amino acid metabolism^27^. Antioxidants are indicated as factors that can maintain the balance between ROS reproduction and clearance. Then, we detected the expression levels of the antioxidant genes in GCs via qRT-PCR between the two groups. In the group of high Gln level, antioxidant gene *IDH1* and glutathione peroxidase 1 (*GPX1*) increased significantly in GCs compared with that in group of low Gln level (P < 0.001 and P < 0.0001, Fig. 4A,B). Moreover, the expression of nuclear factor erythroid 2-related factor 2 (*NRF2*) and its endogenous inhibitor, kelch-like epichlorohydrin-associated protein-1 (*KEAP1*), significantly increased in the high Gln level group (P < 0.01, Fig. 4C,D). Notably, the expression level of *IDH1* mRNA in GCs showed a positive correlation with the Gln concentration in follicular fluid (R = 0.3822, P < 0.05, Fig. 4E). Furthermore, there was a positive correlation between the NE concentration in follicular fluid and *IDH1* expression in GCs (R = 0.4009, P < 0.05, Fig. 4F). To further clarify whether this difference in the expression of antioxidative genes was caused by individual differences, we summarized the clinical data of the IVF patients as Table 2 and found that there were no statistically significant differences in age (P = 0.1601), body mass index (BMI, P = 0.5927), serum Anti-Mullerian Hormone (AMH, P = 0.6033) or thyrotropin (TSH, P = 0.8698) between these two groups. These results demonstrated that the contents of Gln and NE in follicular fluid might increase the antioxidant capacity of GCs.

**Table 2:** Characteristics of patients assessed for follicular fluid analysis.

Characteristics	High level of glutamine	Low level of glutamine	P-value
	(n = 14)	(n = 13)	
Age (years)	31.00[28.75–36.00]	31.00[26.50–33.00]	0.1601
BMI (Kg/m^2^)	21.16[19.52–23.00]	19.95[18.31–21.52]	0.5927
Duration of infertility	2.50[1.00–4.63]	1.00[1.00–3.00]	0.0472
Previous pregnancy	2.00[1.00–3.00]	0[0–1.00]	0.0164
Previous delivery	0[0–0.25]	0[0–0]	0.0820
AMH (ng/ml**)**	2.35[1.29–4.29]	2.90[1.93–3.81]	0.6033
TSH (mIU/L**)**	1.91[1.45–3.03]	2.18[1.83–2.70]	0.8698
Antral follicle count	9.50[5.75–12.25]	9.00[8.50–12.50]	0.9911
**Indication for IVF**			0.3289
Idiopathic	1(7.14%)	1(7.69%)	
Male factor	1(7.14%)	0(0)	
Endometriosis	4(28.58%)	2(15.38%)	
Tube factor	6(42.86%)	6(46.15%)	
Multiple	2(14.28%)	4(30.77%)	

Data are median (interquartile range (IQR)) or numeric value (%).

*AMH* anti-Mullerian hormone.

### IDH1 expression in granulosa cells is positively related with the quality of embryo development of IVF patients

**Figure 5:**
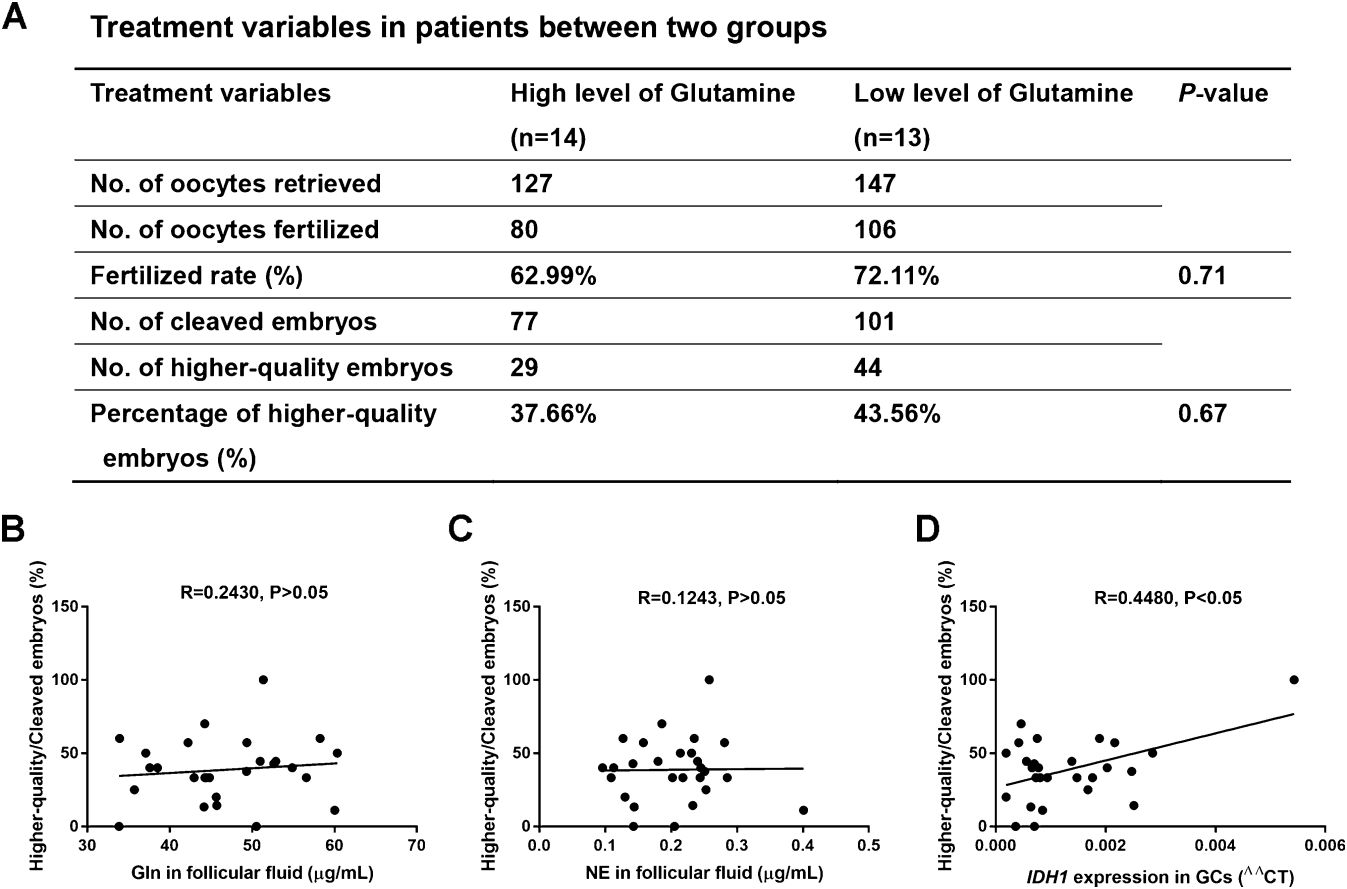
The correlation between glutamine (Gln) or norepinephrine (NE) concentration in follicular fluid and the outcome of in vitro fertilization patients. (**A**) Analysis of the treatment variables in the patients between the two groups. (**B**) Correlation analysis of the Gln concentration in the human follicular fluid and the ratio higher-quality/cleaved embryos (n = 27, R = 0.2430, P > 0.05). (**C**) Correlation analysis of the NE concentration in the human follicular fluid and the ratio of higher-quality/cleaved embryos (n = 27, R = 0.1243, P > 0.05). (**D**) A positive correlation between IDH1 gene expression in cumulus granulosa cells (GCs) and the ratio of higher-quality/cleaved embryos (n = 27, R = 0.4480, P < 0.05).

To determine whether follicular development and oocyte quality were different between the high Gln level and low Gln level groups, we compared several clinical indices in the two groups, including the number of mature follicles (diameters ≥ 14 mm) on the human chorionic gonadotrophin (HCG) injection day, the number of retrieved oocytes, the fertilization rate, and the number and percentage of higher-quality embryos. There were no significant differences between the two groups in the above parameters (P > 0.05, Fig. 5A). Multiple linear regressions were used to identify the relationship between the Gln/NE concentration in follicular fluid and the ratio of higher-quality/cleaved embryos, and IDH1 expression in GCs and the ratio of higher-quality/cleaved embryos after adjusting for spouse age, sperm density, and sperm motility. Results showed that there were no significant correlations between the Gln/NE concentration in follicular fluid and the ratio of higher-quality/cleaved embryos (R = 0.2430 and R = 0.1243, P > 0.05, Fig. 5B,C). However, a significantly positive correlation was observed between IDH1 gene expression in cumulus GCs and the ratio of higher-quality/cleaved embryos (R = 0.4480, P < 0.05, Fig. 5D). These results demonstrated that the IDH1 expression in cumulus GCs may be related with the quality of oocyte and embryo development in IVF patients.

### Glutamine/norepinephrine improve the function of granulosa cells in vitro

**Figure 6:**
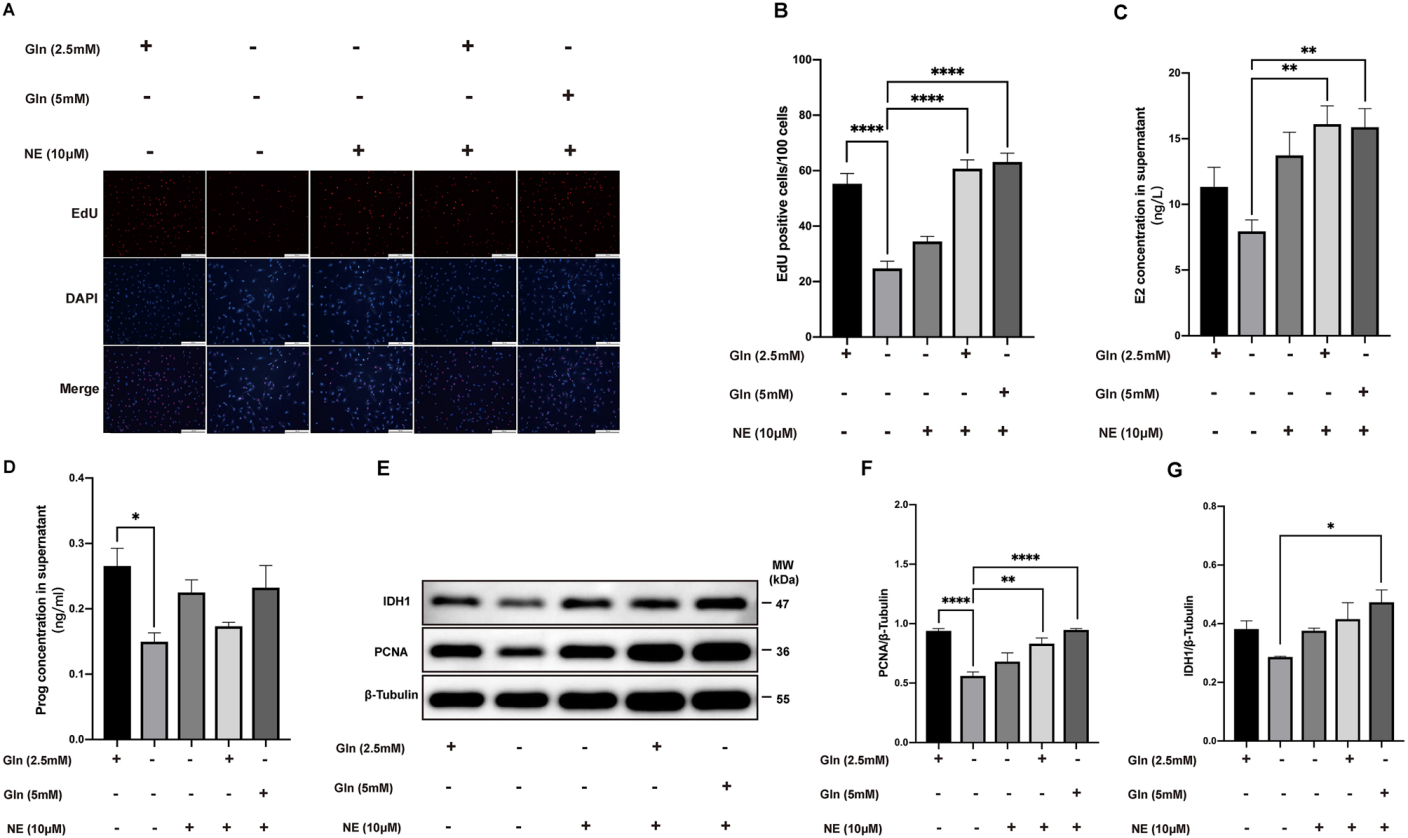
Effect of glutamine (Gln) and norepinephrine (NE) on the function of granulosa cells in vitro (**A**) Immunofluorescent staining of EdU and DAPI in KGN cells for proliferation analysis. (**B**) The column chart shows EdU-positive cells per 100 cells in different treatment groups. (**C**,**D**) Column displays the concentrations of E2 and Prog in the conditioned medium from different treatments of KGN cells. (**E**–**G**) Evaluation of the expression of IDH1 and PCNA protein in different treatment groups by western blotting. All indicated *P* values were determined by one-way analysis of variance. *P value < 0.05; **P value < 0.01; ****P value < 0.0001.

Previous studies have reported that Gln is required for oocyte maturation and embryo development^28^ and adrenergic activity through NE participates in the control of follicular development and steroidal secretion^29^. To study the roles of Gln and NE in GCs, the proliferation of cultured KGN cells in vitro was evaluated via EdU staining and western blotting. Fluorescent staining showed that the percentage of EdU-positive cells was dramatically reduced in the Gln deprivation group. The addition of NE partially increased the ratio of EdU-positive KGN cells. Importantly, Gln supplementation increased significantly the EdU-positive number (P < 0.0001, Fig. 6A,B). Moreover, the concentrations of E2 and Prog in the cell culture supernatant were measured by ELISA. The results showed that the concentration of E2 was reduced in the Gln deprivation group, and adding Gln to the medium significantly increased E2 production in KGN cells, which could be further improved by NE supplementation (P < 0.01, Fig. 6C). Although Gln deprivation reduced the level of Prog in the cell supernatant, Gln and NE supplementation had no obvious effect on Prog production in KGN cells (P < 0.05, Fig. 6D). The expression of PCNA protein in KGN cells exhibited similar trends to the morphological results in the different treatment groups. Notably, Gln deprivation obviously reduced IDH1 protein expression in GCs, which could be recovered by NE and Gln supplementation (P < 0.0001, P < 0.01 and P < 0.05, Fig. 6E–G). These results revealed that the neurotransmitters Gln and NE could synergistically enhance the cell proliferation of GCs and increase the E2 production.

## Discussion

Follicular fluid is an important external environment for the oocyte growth and follicular development. Ideally, follicular fluid, a superfluous, abundant and easily available biological sample in IVF cycles, would be the optimum source of noninvasive prediction of oocyte quality^30^. Currently, investigations of follicular fluid biomarkers for oocyte quality mainly focus on measuring steroid hormones secreted by GCs, such as estradiol and progesterone^31–33^. Therefore, exploring the relationship between metabolites in follicular fluid and granulosa cell function may be conducive to search reliable biomarkers for assessing oocyte quality and predicting the outcome of IVF patients.

Metabolic approach is a powerful tool to analyze the complexities of follicular fluid. Previous study reported the application of gas chromatography-mass spectrometry (GC–MS) to detect polybrominated diphenyl ethers (PBDEs) in follicular fluid and found that the presence of PBDEs could be associated with failed embryo implantation^34^. In the current study, a more simple, fast and efficient LC–MS/MS technology was successfully applied for determination of simultaneous neurotransmitter metabolites in human follicular fluid. Consistent with other studies, we found that many different neurotransmitter metabolites exist in human follicular fluid as shown in Table 1. Among them, high levels of Gln and NE have attracted our attention; there was a positive relationship between the contents of Gln and NE in follicular fluid.

Currently, extensive scientific attention has been given to an important metabolite Gln, which is one of the most abundant amino acid in the human body, such as skeletal muscles, plasma^35^ and follicular fluid^36^. It has anti-inflammatory and antioxidant functions as well as effects that modulate the heat-shock protein response during stress; moreover, as a precursor of glutamate, Gln participates in multiple neurotransmitter metabolic pathways^35,37,38^. In addition, peripheral neurotransmitter NE participates in the control of steroidal secretion from the ovary and follicular development^29^. In this study, we found that the proliferative ability and steroidogenesis of cumulus GCs increased with the high contents of Gln/NE in follicular fluid, which demonstrating that Gln and NE might promote granulosa cell proliferation and up-regulate the expression of steroid hormone syntheses genes.

An altered follicular microenvironment may directly damage follicle recruitment and growth. Previous studies show that metabolites in follicular fluid are direct or indirect regulators of oxidative stress and the function of GCs^39^. Gln as the main energy supply substance for mitochondria to form ATP, is an important source of energy for oocyte maturation and the development of embryo^36^. A study reported that human ovarian tissue was cryopreserved using freezing medium supplemented with cryoprotectants and antioxidants (l-glutamine and taurine) and exhibited good preservation of ovarian vascular integrity and functionality of GCs post thawing^40^. Furthermore, the Gln supplementation could effectively alleviate inflammatory reaction and oxidative stress in PCOS rats^41^. Thus, the relationships among the levels of Gln in follicular fluid, cumulus GC function, and oocyte development are worthy of further study.

Excessive ROS damages DNA, reduces cell proliferation and induces mitochondrial-mediated apoptosis in GCs, which further affect oocyte development^42,43^. Studies have demonstrated that there is a correlation between the apoptosis of GCs and poor oocyte quality and IVF outcomes in patients with PCOS^9,44^. Many environmental stressors may cause inferior oocyte competence and alter oocyte development and growth by disturbing GC function^43,45,46^. In the current study, we found that Gln and NE not only upregulated IDH1 gene expression but also increased GPX1 expression in cumulus GCs. Notably, the concentrations of Gln and NE in follicular fluid were positively related to IDH1 gene expression in cumulus GCs, which could be a predictor of high-quality embryos. The relation of Gln and NE in follicular fluid and antioxidant gene expression in cumulus GCs will provide more valuable information to understand the molecular mechanism of follicular development.

There are several limitations in current study. First, the number of clinical samples was insufficient. Although the number of samples in the two groups met the statistical requirements, the limited number of samples influenced the subsequent analysis of reproductive outcomes of IVF patients. The second limitation was that only antioxidant genes expressions were assayed in cumulus GCs, however, other oxidative stress makers including ROS, MDA, GSH did not detected due to lack of enough samples. Finally, only KGN cells were used to confirm the roles of Gln and NE in maintaining granulosa cell function in vitro experiments. Whether appropriate supplementation of Gln and/or NE in animal models with ovarian dysfunction could improve fertility outcomes needs to be explored in future studies.

## Conclusions

In summary, our research demonstrates that the positive association between Gln/NE content in follicular fluid and the function of human cumulus GCs. Moreover, intrafollicular Gln and NE could upregulate the expression of the antioxidative gene IDH1 in cumulus GCs, which may be a potential predictor for evaluating the quality of embryonic development in the future.

## Supplementary Information

Supplementary Figure.

Supplementary Table.

## Data Availability

The mass spectrometry proteomics data have been deposited to the ProteomeXchange Consortium (http://proteomecentral.proteomexchange.org) via the PRIDE partner repository with the dataset identifier PXD031559 (Username: reviewer_pxd031559@ebi.ac.uk; Password: 5TswXg9B) and PXD031560 (Username: reviewer_pxd031560@ebi.ac.uk; Password: Tfh5K8ts).

## Abbreviations

5-HIAA: 5-Hydroxyindole-3-acetic acid
5-HT: Serotonin hydrochloride
5-HTTP: 5-Hydroxytryptophan
Ach: Acetylcholine chloride
Ala: Alanine
ART: Assisted reproductive technology
AMH: Anti-Mullerian Hormone
BMI: Body mass index
CCND2: Cyclin D2
DA: Hydroxy tyramine hydrochloride
DOPA: Levodopa
E: Adrenaline hydrochloride
E2: 17β-Estradiol
FSHR: Follicle stimulating hormone receptor
GABA: 4-Aminobutyric acid
GCs: Granulosa cells
GC–MS: Gas chromatography-mass spectrometry
GPX1: Glutathione peroxidase 1
Gln: Glutamine
Glu: Glutamate
Gly: Glycine
His: l-Histidine
HisA: Histamine
HCG: Human chorionic gonadotrophin
HPLC: High-performance liquid chromatography
HPLC–MS/MS: High-performance liquid chromatography–mass spectrometry
HSD-3β: Hydroxysteroid dehydrogenase-3beta
HSD-17β: Hydroxysteroid dehydrogenase-17beta
ICSI: Intracytoplasmic sperm injection
IDH1: Isocitrate dehydrogenase 1
IPMCH: International Peace Maternity and Child Health Hospital
IVF: In vitro fertilization
IVF-ET: In vitro fertilization and embryo transfer
*KEAP1*: Kelch-like epichlorohydrin-associated protein-1
Kyn: Kynurenine
KynA: Kynurenic acid
LC–MS/MS: Liquid chromatography–tandem mass spectrometry
LHR: Luteinizing hormone receptor
CIs: Confidence intervals
MRM: Multiple reaction monitoring
MT: Melatonin
NE: Norepinephrine
NRF2: Nuclear factor erythroid 2-related factor 2
OCC: Oocyte cumulus complex
PA: Picolinic acid
PBDEs: Polybrominated diphenyl ethers
PCNA: Proliferating cell nuclear antigen
PCOS: Polycystic ovary syndrome
Pro: Proline
Prog: Progesterone
ROS: Reactive oxygen species
STAR: Steroid acute regulatory protein
Trp: Tryptophan
TrpA: Tryptamine
TSH: Thyrotropin
Tyr: Tyrosine
TyrA: Tyramine
XA: Xanthurenic acid
WHO: World Health Organization
VWA: Vanillylmandelic Acid
