# Supplementary material for: Glutamine and norepinephrine in follicular fluid synergistically enhance the antioxidant capacity of human granulosa cells and the outcome of IVF-ET
Source: Sci Rep. 2022 Jun 15;12:9936. doi: 10.1038/s41598-022-14201-1 (PMC9200745; doi:10.1038/s41598-022-14201-1)
Supplement: Supplementary file 1 — Supplementary Figure. [file 41598_2022_14201_MOESM1_ESM.pdf]

Supporting Information

Glutamine and norepinephrine in follicular fluid synergistically enhance the antioxidant capacity of human granulosa cells and the outcome of IVF-ET

Lulu Wang[#1], Chengliang Zhou[#1], Junyan Sun[1], Qiuwan Zhang*[1,2], Dongmei Lai*[1,2]

[1.]The International Peace Maternity and Child Health Hospital, School of Medicine, Shanghai Jiao Tong University; [2.]Shanghai Key Laboratory of Embryo Original Diseases; Shanghai 200030, China

[#]Lulu Wang and Chengliang Zhou contributed equally to this work

*Corresponding Author:

Dongmei Lai, MD, PhD

E-mail: laidongmei@hotmail.com

Qiuwan Zhang, PhD

E-mail: zhangqiuwan@163.com

145, Guang-Yuan Road, Shanghai 200030, P. R. China, Tel: 86-21-64070434,

Fax: +86-21-64074642

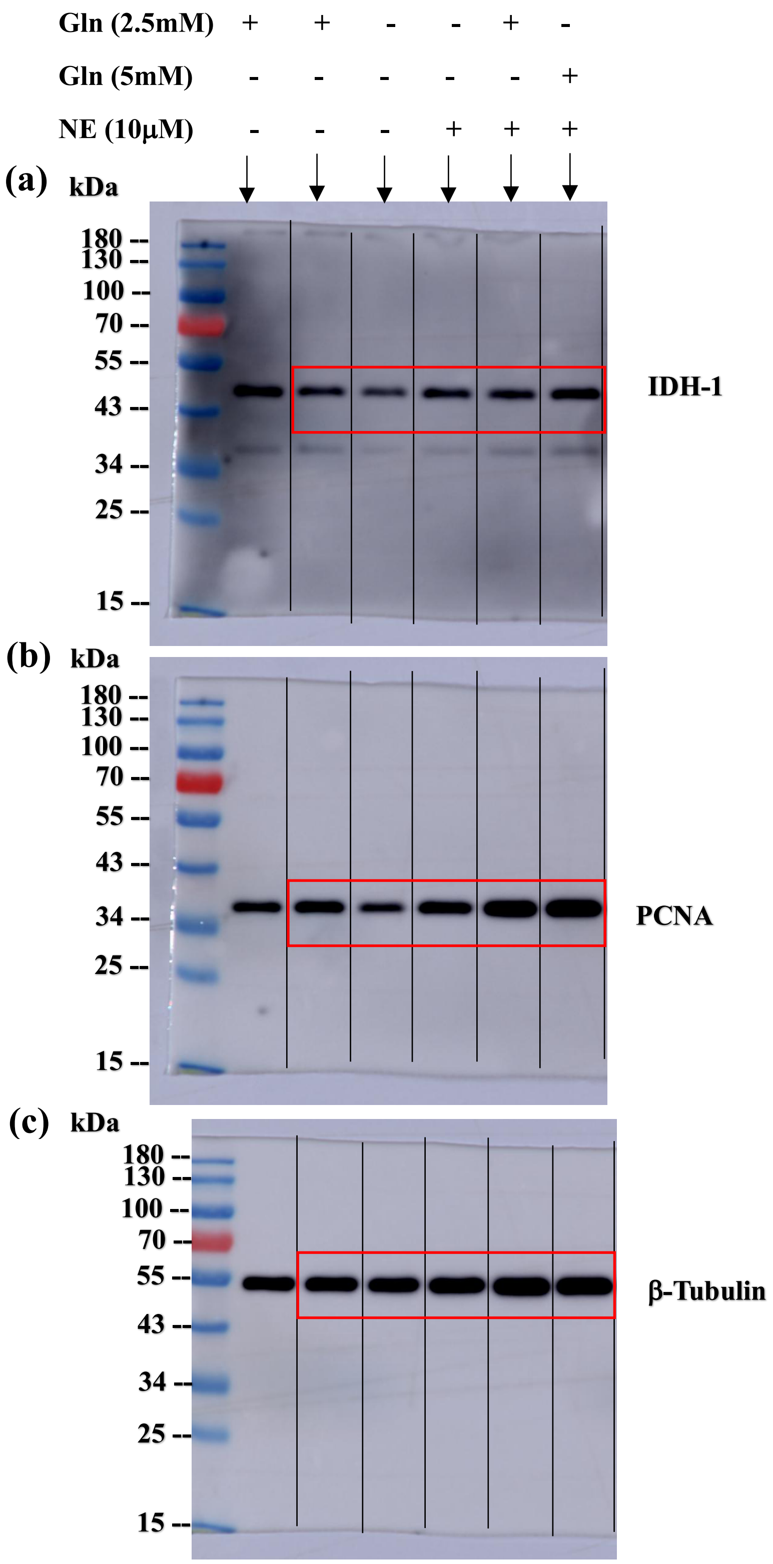

Figure S1. (a) - (c) The full-length blot images showing the grouping of blots in Figure 6E were from the same gel.

Molecular weight marker for protein (kDa) indicated. Red frames indicate cropped parts used in figure 6E.
