# Supplementary material for: Glutamine and norepinephrine in follicular fluid synergistically enhance the antioxidant capacity of human granulosa cells and the outcome of IVF-ET
Source: Sci Rep. 2022 Jun 15;12:9936. doi: 10.1038/s41598-022-14201-1 (PMC9200745; doi:10.1038/s41598-022-14201-1)
Supplement: Supplementary file 2 — Supplementary Table. [file 41598_2022_14201_MOESM2_ESM.docx]

| **Supplementary Table 1. Primer sequences for qRT-PCR in human granulosa cells** | | |
| --- | --- | --- |
| **Target genes** | **Primer sequence(5'-3')** | **Product Size (bp)** |
| IDH1 | F: AGAAGCATAATGTTGGCGTCA | 106 bp |
|  | R: CGTATGGTGCCATTTGGTGATT |  |
| GPX1 | F: CCGGGACTACACCCAGATGA | 101 bp |
|  | R: CGTTCTCCTGATGCCCAAAC |  |
| NRF2 | F: TCAGCGACGGAAAGAGTATGA | 174 bp |
|  | R: CCACTGGTTTCTGACTGGATGT |  |
| KEAP1 | F: CTGGAGGATCATACCAAGCAGG | 220 bp |
|  | R: GGATACCCTCAATGGACACCAC |  |
| PCNA | F: CCTGCTGGGATATTAGCTCCA | 109 bp |
|  | R: CAGCGGTAGGTGTCGAAGC |  |
| CCND2 | F: TTTGCCATGTACCCACCGTC | 104 bp |
|  | R: AGGGCATCACAAGTGAGCG |  |
| LHR | F: CACATAACCACCATACCAGGAAA | 124/283 bp |
|  | R: AAGTCAGTGTCGTCCCATTGA |  |
| FSHR | F: AAAGCTGCCTACTCTGGAAAAG | 170 bp |
|  | R: GACCCCTAGCCTGAGTCATATAA |  |
| STAR | F: GTGGAACCCCAGTGTCAAGA | 129 bp |
|  | R: CAGCGCACACTCACAAAGTC |  |
| CYP11A | F: GAGGCCCAGCGATTCATTGAT | 94 bp |
|  | R: TCCTGAACAGACGGAACAGGT |  |
| CYP19A | F: TGGAAATGCTGAACCCGATAC | 161 bp |
|  | R: AATTCCCATGCAGTAGCCAGG |  |
| HSD-3β | F: CTTGTGCGTTAAGACCCACAT | 124 bp |
|  | R: GGGTTGACTGTAGAGAACTTTCC |  |
| HSD-17β | F: GTGCTGGTGTGTAACGCAG | 98 bp |
|  | R: GTCCCTACTACATTCACGTCCA |  |
| 18sRNA | F: GTAACCCGTTGAACCCCATT | 151 bp |
|  | R: CCATCCAATCGGTAGTAGCG |  |
